# Mussel Consumption as a “Food First” Approach to Improve Omega-3 Status

**DOI:** 10.3390/nu11061381

**Published:** 2019-06-19

**Authors:** Stefano Carboni, Gunveen Kaur, Abigail Pryce, Kyle McKee, Andrew P. Desbois, James R. Dick, Stuart D. R. Galloway, David Lee Hamilton

**Affiliations:** 1 Faculty of Natural Sciences Institute of Aquaculture, University of Stirling, Pathfoot Building, Stirling, FK9 4LA, UK; stefano.carboni@stir.ac.uk (S.C.); andrew.desbois@stir.ac.uk (A.P.D.); j.r.dick@stir.ac.uk (J.R.D.); 2 Institute for Physical Activity and Nutrition (IPAN), School of Exercise and Nutrition Sciences, Deakin University, Geelong 3216, Australia; gunveen.kaur@deakin.edu.au; 3 Faculty of Health Sciences and Sport, Physiology, Exercise and Nutrition Research Group, University of Stirling, Cottrell Building, Stirling, FK9 4LA, UK; apryce202@caledonian.ac.uk (A.P.), KyleM0419@live.com (K.M.); s.d.r.galloway@stir.ac.uk (S.D.R.G.)

**Keywords:** mussels, *Mytilus edulis*, omega-3 fatty acids, omega-3 index, nutrition, n-3 polyunsaturated fatty acids (PUFAs), eicosapentaenoic acid (EPA), docosahexaenoic acid (DHA), sustainability

## Abstract

Numerous United Kingdom and European Union expert panels recommend that the general adult population consumes ~250 mg of eicosapentaenoic acid (EPA) and docosahexaenoic acid (DHA) per day through the consumption of one portion of oily fish per week. The long-chain omega-3 fatty acids EPA and DHA are only found in appreciable amounts in marine organisms. Increasing oily fish consumption conflicts with sustaining fisheries, so alternative dietary sources of EPA and DHA must be explored. Mussels are high in omega-3 polyunsaturated fatty acids (PUFAs) and a good source of essential amino acids. Therefore, we aimed to investigate the impact of introducing mussels as a protein source in the lunchtime meal three times per week for two weeks on the omega-3 status of free-living participants. Following an initial two-week monitoring period, 12 participants (eight male and four female) attended the nutrition laboratory three times per week for two weeks. Each participant received a personalised lunch constituting one-third of their typical daily calorie consumption with ~20% of the calories supplied as cooked mussels. A portion of cooked mussels from each feeding occasion was tested for total omega-3 content. The mean ± SD mussel EPA + DHA content was 518.9 ± 155.7 mg/100 g cooked weight, meaning that each participant received on average 709.2 ± 252.6 mg of EPA + DHA per meal or 304.0 ± 108.2 mg of EPA + DHA per day. Blood spot analysis revealed a significant increase in the omega-3 index (week 1 = 4.27 ± 0.81; week 4 = 5.07 ± 1.00) and whole blood EPA content during the study (%EPA week 1 = 0.70 ± 0.0.35; %EPA week 4 = 0.98 ± 0.35). Consuming mussels three times per week for two weeks as the protein source in a personalised lunchtime meal is sufficient to moderately improve the omega-3 index and whole blood DHA + EPA content in young healthy adults.

## 1. Introduction

The European Food Safety Authority (EFSA) has set the adequate combined intake for the marine-based long-chain omega-3 fatty acids, eicosapentaenoic acid (EPA) and docosahexaenoic acid (DHA), as 250 mg/day [1]. The National Health Service (NHS) in the U.K. recommends that the general adult population achieve this intake target through the consumption of one portion (~140 g/portion) of oily fish per week [2]. However, it is estimated that less than 20% of the global population achieves this intake [3], with mean U.K. intake estimated at approximately one-third of a portion per week [4]. Given the potential health benefits of EPA and DHA [5], the advice to the general adult population is to increase consumption of oily fish, but this advice conflicts with achieving and maintaining sustainable fisheries. If current trends continue, it is estimated that fish stocks will be irreversibly damaged within the next few decades [6]. The general public seem to be disproportionately concerned with the toxin content of oily fish rather than the nutrient content [7], possibly resulting in reluctance to increase consumption. As a result of the conflicts between the advice to consume more oily fish, public concern over toxin content, and the negative impact that increasing consumption would have on marine biodiversity, it is crucial that we explore alternative food sources of the long-chain omega-3 fatty acids, EPA and DHA. One such source is shellfish and, in particular, mussels.

Mussels are an environmentally sustainable way of producing dietary protein and long-chain omega-3 fatty acids. The greenhouse gas (GHG) emissions produced from farming Scottish Mussels (*Mytilus edulis* L.) are a fraction of those from producing salmon or terrestrial meat [8]. Whereas the environmental impact of mussel farming is complex, it does have the potential to positively impact aquaculture environments [9]. Mussel farming does not affect the sustainability of marine fisheries and although mussels may present an allergy and food poisoning risk [10], the contaminant content of Scottish farmed mussels is well below the European Union (EU) limits [11] and, in most cases, is well below the levels seen in fish caught around the coast of the U.K. [12]. Therefore, encouraging the population to increase consumption of mussels may be a viable strategy to improve the intake of EPA and DHA without the same concerns about toxins and environmental damage.

Not only are mussels a sustainable source of omega-3 fatty acids, they are also a sustainable source of essential amino acids [10,13,14]. The protein content in mussels varies between 12.6 and 24.0 g/100 g mussels, depending on the variety [10]. Mussels contain a range of vitamins and minerals found in other meat-based sources of protein such as B-vitamins and trace minerals [10]. As a result, replacing the protein portion of selected meals throughout the week with a portion of mussels may be a viable strategy to improve omega-3 status and nutrient sufficiency amongst the general adult population without negatively impacting marine biodiversity. To avoid the risk of inadvertent doping through contaminated supplements, the Sports and Exercise Nutrition register (SENr) suggests in their position stand that a “food first” approach should be sought wherever possible to enhance the intake of specific nutrients [15]. Achieving improvements in omeaga-3 status without the use of supplements presents a challenge considering the NHS recommends no more than four portions of oily fish be consumed per week [2]. Despite the breadth of data demonstrating the impact of omega-3 supplementation on omega-3 status [16,17], comparatively little data address the impact of eating omega-3-containing foods on omega-3 status in the context of a controlled trial. Supplementation with omega-3 fatty acids from capsules or an equivalent dose from food leads to very similar changes in omega-3 status [18], but the predominance of fatty acids incorporated into the blood or tissues is dependent upon the predominant fatty acid supplied [16,19]. Mussels are relatively high in EPA and DHA [10] and should therefore be effective at improving EPA and DHA status; however, to the best of our knowledge, no study has addressed the impact of consuming mussels on omega-3 status in free living humans. As there is no upper limit for the safe consumption of shellfish [2], mussels may be an effective food first approach to safely increase omega-3 status. Therefore, the aim of this study was to assess if using locally-sourced mussels as the protein component of lunchtime meals three times per week for two weeks would be sufficient to modify the omega-3 status, as assessed by the omega-3 index (blood EPA + DHA content as described by Harris and Von Schacky [20]), and whole blood omega-3 content, in young, healthy, free-living volunteers.

## 2. Methods

### 2.1. Participants

This study was approved by the Faculty of Health Sciences and Sport Research Ethics Committee. All participants provided informed, written consent prior to taking part in the study. Participant data were protected in accord with the guidelines of the Data Protection Act [21]. All testing occurred in the Nutrition or Resting Laboratories at the University of Stirling. The participants of this study were recruited from staff and students at the University of Stirling. In total, 15 individuals provided informed written consent to participate in the study with three dropouts (one case of illness, two dropped out due to not liking mussels), resulting in a total of 12 participants (8 men and 4 women). Participant characteristics are presented in Table 1. Given the purpose of the study, participants were required to not regularly meet the recommended daily intake of oily fish/omega-3 or be taking any kind of omega-3 supplement. Exclusion criteria prohibited any participants suffering from a shellfish or a similar allergy from participating. Recruitment was achieved through posters positioned around the University and via word of mouth.

### 2.2. Diet Analysis and Blood Sampling

Participant dietary intake was recorded using 3 days/week food diaries. Participants were provided with scales to weigh food accurately. Food diary data were input and analysed by nutritional software (Nutritics Academic Edition v4.267; Nutritics, Dublin, Ireland). Scales and a stadiometer were used to measure weight (kg) and height (cm), respectively. Blood samples were taken via finger prick using sterile lances. The blood was then collected on cards pre-treated with butylated hydroxytoluene (BHT; 50 mg/100 mL in ethanol). Cards were stored in clean/dry Tupperware tubs with desiccant before being transferred to the desiccator to dry each sample to completion. Blood samples were collected in duplicate in a rested, fasted state.

### 2.3. Design

The experimental design used for this study was a repeated measures model, with each participant undergoing two baseline-testing days on two consecutive weeks (W1 and W2) to demonstrate the stability of their blood analysis, followed by six feeding occasions on non-consecutive days over the next two weeks (W3 and W4).

On day 1 in Week 1 (W1), participants had their height and weight measured and provided a blood sample via finger prick. They were asked to fill in a food preference questionnaire so that the meals provided in the testing portion of the study were suited to tastes. Participants also received 3 days/week food diaries and food scales to record food consumption over the course of the 4-week study. Participants were asked to maintain their habitual activity levels throughout the study. On day 1 in Week 2 (W2), participants again had their height and weight assessed and handed over their recorded food diary to allow calculation of the caloric and macronutrient requirements for their test meals. They provided a second finger prick blood sample and were given another 3 days/week food diary. Subjects were asked to attend the laboratory on three non-consecutive days for the following 2 weeks (W3 and W4) to receive their test meals (see Table 2 for the macronutrient breakdown and Table 3 for the recipes).

A finger prick blood sample was then taken on the Monday morning following each feeding week. Blood samples via finger prick were always taken in the morning while participants were in an overnight fasted state. Weight was measured each time participants entered the laboratory for blood sampling to ensure stable weight during the course of the study (W1, W2, W3, and W4). Food diaries were handed in on the first day of W2, W3, and W4. Finally, in W3 and W4, participants received meals containing mussels as the protein component of the meal 3 times per week at lunchtime, individualised to the food diaries completed in W1.

As discussed above, the test meals were tailored to the individual participant dependent upon the results of their food diary analysis. Baseline food diaries were analysed in Nutritics (Nutritics Academic Edition v4.267; Nutritics, Dublin, Ireland), and from this, the meals were designed to contain one-third of each participant’s typical daily calorie intake. The macronutrient split was as follows: 60% carbohydrate (CHO), 20% fat, and 20% protein (PRO). Every participant received a minimum of 80 g of cooked mussels. Meals were provided on Monday, Wednesday, and Friday. They were fed at lunchtime in one of two self-selected feeding slots, either 12:00 p.m. or 1:00 p.m.

### 2.4. Macronutrient Calculations

The macronutrient range was set within the acceptable macronutrient distribution ranges (AMDRs): 60% CHO, 20% fat, and 20% PRO. These AMDR values were selected so that each participant received an adequate dose of ~20 g PRO in each test meal.

The data in Table 2 were based on calorie intake from W1 food diary analysis. Analysis was performed using Nutritics (Nutritics Academic Edition v4.267; Nutritics, Dublin, Ireland). Total intake was divided by 3 to calculate the lunch total. Lunch total was then reported as energy content of each macronutrient (kcals) with the meal composition consisting of 60% CHO, 20% fat, and 20% PRO, with gram intake calculated using Atwater factors of 4, 9, and 4 kcal/g, respectively. These values were used to calculate the breakdown of the meals each participant received.

### 2.5. Mussel Preparation 

Prior to cooking, mussels were checked for viability, cleaned, beards removed, and cooked before participants entered the laboratory. Mussels were steamed in a large pot for approximately 8 minutes until shells opened. Only mussels that had fully opened were served to participants. Closed-shell mussels were discarded. Mussel meat was then de-shelled before being provided to participants for consumption. Mussels were prepared in accordance with recommendations laid out by the U.K. Food Standards Agency (FSA) [22]. Subjects were asked to eat all the food served to them, and water was provided ad libitum.

### 2.6. Baseline Measurements

Baseline measurements of height (m) and weight (kg) were taken using a standardised method outlined by National Health and Nutritional Examination Survey (NHANES) Anthropometry Procedures Manual [23]. The same set of scales was used at each weigh-in. Measurements were recorded twice and a mean was obtained. Weight was measured each week to record the stability of body weight throughout the study. Height was measured once in the first week so that a body mass index (BMI) for each participant could be calculated.

Blood was collected via finger prick. Participants were asked to thoroughly wash hands with soap and warm water and to dry hands completely prior to finger prick. Next, each participant’s non-dominant hand middle-finger was cleaned with an anti-bacterial wipe and a lancet was used to draw capillary blood.

### 2.7. Blood Spot Analysis

Blood spots arrived at the laboratory and were dried fully under vacuum. An automated method was used for analysis of blood spots. A CTC PAL HTX-*xt* robot (CTC Analytics AG, Zwingen, Switzerland) was used to extract lipid from blood spots and purify and prepare fatty acid methyl esters (FAME). The blood spot was cut out and placed in a 10-mL screw cap sample vial and loaded into the machine carousel. The robot then conduced the following process: 1 mL of 1.25 M HCl in methanol was added, heated to 70 °C for 1 h, and then cooled to room temperature. Then, 3 mL iso-hexane containing of 0.01% (*w/v*) BHT and 4 mL saturated KCl solution was added. This mixture was shaken for 4 minutes in an agitator and allowed to settle for 2 minutes. Next, 2.5 mL of the top organic phase was passed through a pre-conditioned solid-phase extraction cartridge (washed with 5 mL iso-hexane). FAME were eluted with 5 mL iso-hexane/diethyl ether (95:5, *v/v*). Then, the solvent from each vial was removed by evaporation under nitrogen, the FAME re-dissolved in 200 µL iso-hexane, and then transferred to an autosampler vial for gas-liquid chromatography (GLC) analysis as described in Bell et al. [24]. The omega-3 index was calculated as described by Harris and Von Schaky [20]. The percentage of highly-unsaturated fatty acids (HUFA) represents the percentage of omega-3 fatty acids in the HUFA fraction as described by Lands and Lamoreaux [25].

### 2.8. FAME Analysis of Mussel Samples

A portion of cooked mussels from each feeding occasion was retained and frozen at −80 °C for later analysis of the FAME profile. Total lipids were extracted by homogenising in 20 volumes of chloroform/methanol (2:1, *v/v*). Total lipids were prepared according to the method of Folch et al. [26] and non-lipid impurities were removed by washing with 0.88% (*w/v*) KCl. The weight of lipids was determined gravimetrically after evaporation of solvent and overnight desiccation under vacuum. FAME were prepared by acid-catalysed transesterification of total lipids according to the method of Christie et al. [27]. Extraction and purification of FAME was performed as described by Ghioni et al. [28]. FAME were separated by GLC using a ThermoFisher Trace GC 2000 (Hemel Hempstead, U.K.) equipped with a fused silica capillary column (ZBWax, 60 m × 0.25 μm × 0.25 mm i.d.; Phenomenex, Macclesfield, U.K.) with hydrogen as the carrier gas and using on-column injection. The temperature gradient ranged from 50 to 150 °C at 40 °C/min and then to 195 °C at 1.5 °C/min and finally to 220 °C at 2 °C/min. Individual FAME were identified by reference to published data [29]. Data were collected and processed using the Chromcard for Windows (version 2.00) computer package (Thermoquest Italia S.p.A., Milan, Italy).

### 2.9. Statistical Analysis

All data are presented as mean ± standard error, unless stated otherwise, with individual data points also presented. Time course analysis was conducted with a one-way ANOVA followed by a Tukey’s Honest Significance Different test; bars not connected by the same letters are significantly different from each other.

## 3. Results

### 3.1. Subject Characteristics and Diet

A total of 15 participants were originally recruited to the study (nine men, six women). However, of those, only 12 participants (eight men, four women) completed the four weeks of the trial and attended all six feeding sessions. One subject dropped out due to illness. Two other subjects dropped out after the first test meal citing distaste for mussels as the reason for discontinuing the study. One subject (male) was removed from analysis due to reporting a high intake of tinned mackerel on the run up to the study leading to a very high baseline omega-3 index. All participants were university students in their mid–early 20 s (23.9 ± 1.4 years) and had a healthy BMI (24.88 ± 0.74 kg.m^−2^) (Table 1). The diet characteristics and the macronutrient composition of each subject’s test meals are reported in Table 2. Subjects’ weighed food diet diaries indicated a mean daily caloric intake of 2144 ± 273.8 kcal/day. Table 3 shows the recipe breakdown of each of the three test meals. Test meals were designed to be palatable to all subjects based on the results of the diet preference questionnaires.

### 3.2. Mussel Omega-3 Content

**Figure 1:**
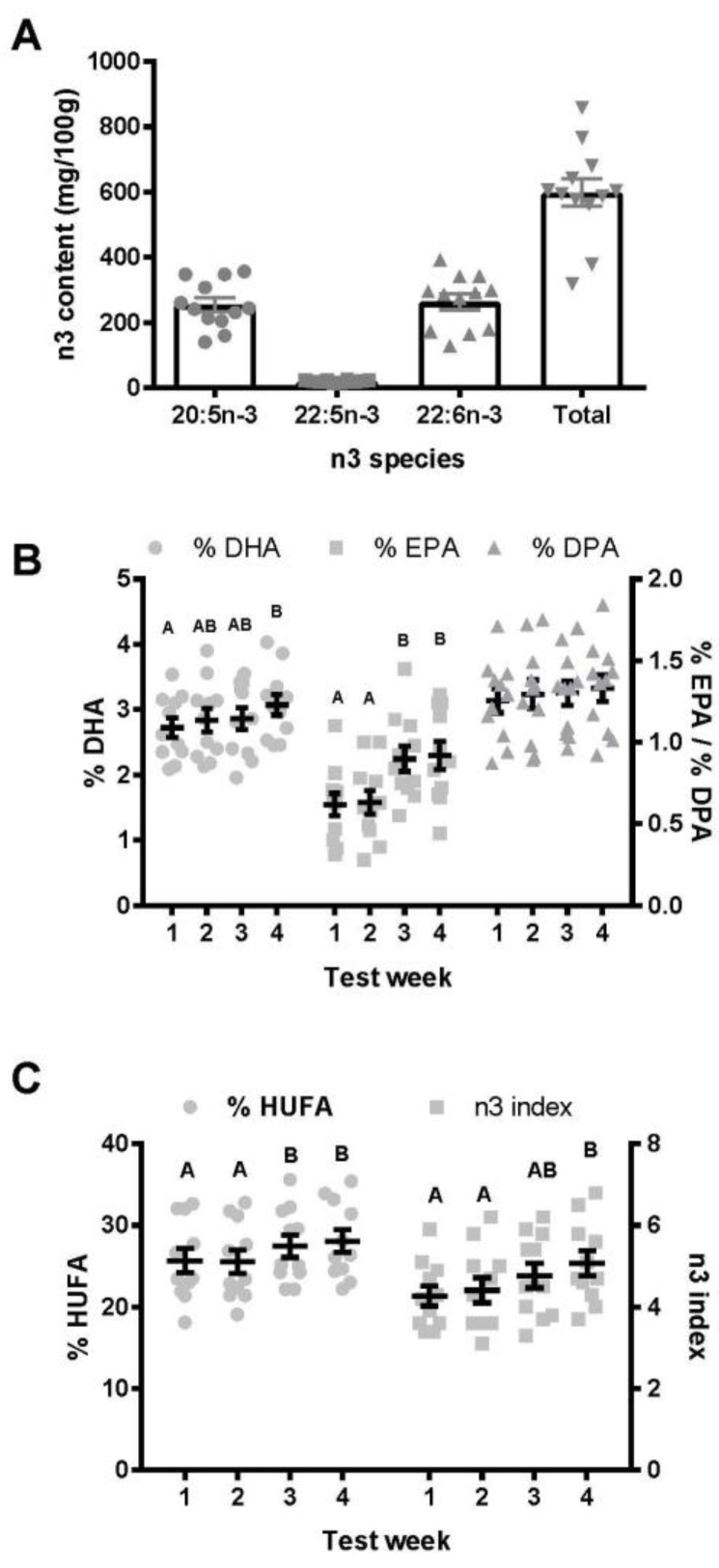
(**A**) Mussel omega-3 fatty acid content, (**B**) percentage of fatty acids as EPA, DPA, and DHA in whole blood, (**C**) percent of HUFA fatty acids that contain n-3 fatty acids and n-3 Index. Individual data points plotted with mean ± SEM represented as bars in (A) or lines in (B and C). Data not connected by the same letter are significantly different (*p* < 0.05).

Over the course of the trial, there were 24 feeding occasions from 12 separate batches of locally sourced Scottish mussels. Each batch of cooked mussels was sampled and frozen at −80°C for subsequent analysis of the fatty acid composition. The mean total omega-3 content (Figure 1A) was 598.3 ± 41.96 mg/100g, with EPA and DHA each representing just over 40% of the total (EPA = 42%, DHA = 44%, docosapentaenoic acid (DPA) = 3.4%) omega-3 content. There was considerable variation from batch to batch with a minimum omega-3 content of 319.6 mg/100 g cooked meat and a maximum of 858.8 mg/100 g cooked meat. Over the course of the six feeding occasions, each subject consumed a mean of 709.2 ± 252.6 mg of EPA + DHA per meal or 304.0 ± 108.2 mg of EPA + DHA per day, meaning that our feeding paradigm achieved a mean intake slightly above the EFSA recommended intake of 250 mg of combined EPA + DHA per day.

### 3.3. Whole Blood Omega-3 Levels and Omega-3 Index

As this was a repeated measures design without a control group, we introduced an additional week of monitoring to ensure the stability of the blood measures prior to introducing the test meals. During the two monitoring weeks (W1 and W2), there were no significant differences in any of the blood measures assessed and Pearson’s correlation coefficient (*r*) was close to one in all cases, indicating a high degree of reliability from W1 to W2 (Figure 1B,C). For instance, from W1 to W2, the Pearson’s correlation coefficient for %EPA, %DHA, omega-3 index, and %HUFA were 0.90, 0.90, 0.90, and 0.99, respectively. The fold-change variations from W1 to W2 for %EPA, %DHA, omega-3 index, and %HUFA were 0.13, 0.058, 0.068, and 0.025, respectively. %DPA did not significantly change from W1 to W4.

To ensure that any changes in blood omega-3 content were due to the stable incorporation of omega-3 fatty acids into the blood rather than an acute effect of meal feeding, the blood spots analysis were collected on the Monday morning in an overnight fasted state after the previous week’s test meals (last feeding session being lunchtime on Friday). %EPA significantly (*p* ≤ 0.01) increased from W1 (0.69 ± 0.10%) and W2 (0.68 ± 0.08%) to W3 (0.95 ± 0.09%) and W4 (0.98 ± 0.10%), with an overall W1 to W4 effect size of 1.20 (a 1.535 ± 0.088-fold increase; W1 to W2 variance = 0.129-fold) (Figure 1B). For %DHA, there was a significant (*p* ≤ 0.01) increase from W1 (2.75 ± 0.15%) to W4 (3.07 ± 0.16%), with an overall W1 to W4 effect size of 0.64 (a 1.13 ± 0.032-fold increase; W1 to W2 variance = 0.058-fold) (Figure 1B). For %DPA, there was no significant change across the four weeks. For the omega-3 index, there was a significant (*p* ≤ 0.01) increase from W1 (4.27 ± 0.24) and W2 (4.41 ± 0.30%) to W4 (5.07 ± 0.30%), with an overall W1 to W4 effect size of 0.84 (a 1.191 ± 0.034-fold increase; W1 to W2 variance = 0.068-fold) (Figure 1C). For %HUFA, there was a significant (*p* ≤ 0.02) increase from W1 (25.69 ± 1.48%) and W2 (25.53 ± 1.43%) to W3 (27.43 ± 1.36%) and W4 (28.07 ± 1.41%), with an overall W1 to W4 effect size of 0.51 (a 1.099 ± 0.02-fold increase; W1 to W2 variance = 0.025-fold) (Figure 1C).

For all measures reported, the increases from W1 to W4 were approximately three times or more than the variance reported from W1 to W2, indicating that the significant changes reported were statistically meaningful.

**Figure 2:**
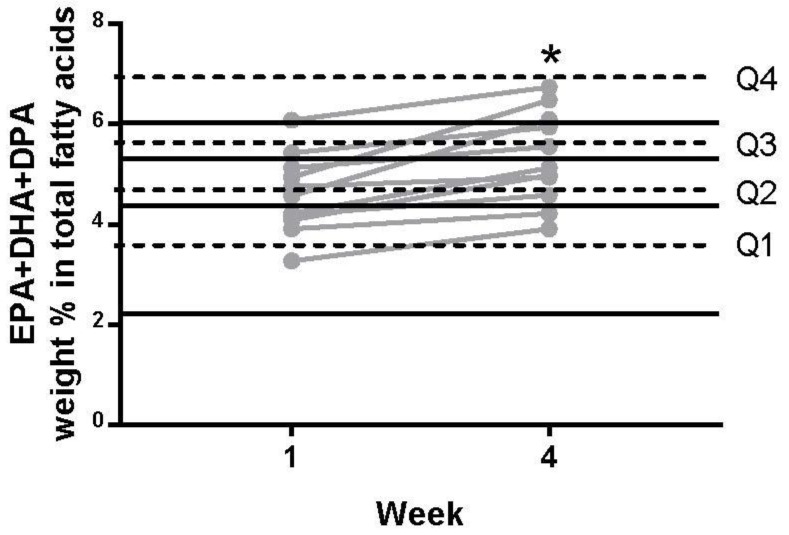
Percentage weight of whole blood fatty acids as combined EPA, DHA, and DPA represented as individual data points in W1 and W4. Each subject’s data are connected by a grey line. * indicates significantly different from W1 (*p* < 0.05). Quartiles for cardiac risk were taken from the literature [17,30].

To assess the clinical relevance of the above changes, we plotted the W1 to W4 changes in the %EPA + %DHA + %DPA content within the sudden cardiac death risk quartiles (Figure 2) reported by Albert et al. and Patterson et al. [17,30]. Six of the 11 subjects shifted up one or two quartiles following two weeks of consuming mussels. Three subjects moved from quartile 1 to 2, corresponding to a 45% reduction in the risk of sudden cardiac death. Two subjects moved from quartile 2 to quartile 3, indicating a ~25% reduction in the risk of sudden cardiac death. One of the subjects moved up two quartiles from quartile 2 to quartile 4, indicating a ~35% reduction in the risk of sudden cardiac death. Of the subjects who did not cross a risk quartile, two remained in quartile 4, one remained in quartile 2, one remained in quartile 3 and one in quartile 4.

## 4. Discussion

The health benefits of consuming supplemental long-chain omega-3 fatty acids have been well researched [5]. Less well researched, however, are food first approaches to enhancing omega-3 status in an environmentally sustainable manner. Several studies have shown that incorporating fish into the diet of patients with cardiovascular disease can enhance omega-3 status and improve blood lipid profiles [31]. However, current evidence suggests that the omega-3 fatty acids in the world fish stocks ae insufficient to meet the population’s daily requirement for omega-3 fatty acids [4]. Therefore, sustainable sources of omega-3 fatty acids that do not impact marine biodiversity need to be investigated. Mussels are one such potential source [10]. To the best of our knowledge, this study is the first to use mussels in a food first manner to sustainably enhance omega-3 status. We showed, for the first time, that when mussels are supplied as the protein component of lunchtime meals in a manner individualised to each subject’s dietary needs, omega-3 status is significantly improved. When compared against clinically relevant outcomes, such as risk of sudden cardiac death, 6 of the 11 subjects improved their omega-3 status to a degree that is associated with at least a 20% reduction in sudden cardiac death risk.

Compared with other food-based sources of long-chain omega-3 fatty acids, mussels have a relatively high content of the long-chain omega-3 fatty acids, EPA and DHA [10]. The mean omega-3 content of mussels in our study is similar to previously published results [10]. However, we did note an almost three-fold variation in omega-3 content from batch to batch, indicating that omega-3 intake from mussels may not always be representative of published norms. This can potentially be attributed to the seasonal variation linked to the reproductive cycle and to geographical differences in microalgae species composition in the different farming sites [32,33,34,35]. In addition to long-chain omega-3 fatty acids, mussels are also a good source of protein and a range of other vitamins and minerals [10,13,14]. Consequently, regularly including mussels in the diet could be a sustainable way to achieve nutritional sufficiency if they are used to replace other protein sources such as pork, beef, or chicken. The GHG emissions per edible kilogram of mussels are a fraction of that from producing pork, beef, or chicken [8]. Harvesting rope-cultured mussels is unlikely to impact the marine biodiversity in the same way as commercial scale fishing [9]. As a result, mussels could be a highly nutritious, omega-3-containing protein source for environmentally conscious consumers.

Although we found that mussels contain relatively high amounts of EPA and DHA, they appeared to have a very low content of DPA. Whereas both EPA and DHA measures in our subjects were significantly improved by W4 of our study, DPA status did not significantly change. Our previous work has shown that supplementing cultured muscle cells [36] or rodents [37] with EPA leads to significant improvements in DPA in cultured muscle and rodent liver, respectively. Supplementing humans with an EPA/DHA supplement improves blood DPA levels by approximately one-third after two weeks of supplementation [38]. However, supplementing humans with EPA alone for seven days has no effect on DPA status [39]. In our present study, despite substantially improving the EPA status of our subjects, DPA status remained unchanged. As DPA has potential bioactivity [40] in its own right, different from that of EPA or DHA, it may be important to consume other sources of long-chain omega-3 fatty acids to ensure the DPA status also improves.

To conclude, replacing the protein component of lunchtime meals three times per week for two weeks with mussels is sufficient to moderately improve omega-3 status. For some subjects, the improvements in omega-3 status were of a magnitude that is clinically relevant, corresponding to a reduction in the risk of sudden cardiac death. Although an apparently good source of EPA and DHA, mussels appear to be a poor source of DPA, so other feeding strategies would be required to enhance DPA status. Further research should examine the time-course and dose-response effects of feeding mussels to determine how long or how much more mussel meat would be required to enhance omega-3 status further. Finally, because we had a 14% dropout rate due to distaste for mussels, it would be pertinent (while being mindful of allergy risk) to explore methods of concealing mussels in food stuffs to ensure that the health and environmental benefits of consuming mussels could be shared by as much of the population as possible.

## Figures and Tables

**Table 1:** Descriptive information on the study participants.

Parameter	Descriptive (*n* = 12)
Age (years)	23.9 ± 1.3
Body mass (kg)	77.4 ± 3.5
Stature (m)	1.76 ± 0.03
Body mass index (kg/m^2^)	24.88 ± 0.74

Note: Values expressed as mean ± standard error of the mean (SEM).

**Table 2:** Individual breakdown of energy and macronutrient intake for each test meal.

Participant	Daily Energy (kcals)	Lunch Total (kcals)	Protein (kcals)	Protein (g)	CHO (kcals)	CHO (g)	Fat (kcals)	Fat (g)
1	1272	424	85	21	254	64	85	9
2	1470	490	98	25	294	74	122	14
3	1824	608	122	30	365	91	152	17
4	1167	389	78	20	233	58	97	11
5	2509	836	167	42	502	125	209	23
6	4174	1391	278	70	835	208	348	39
7	2624	874	175	44	525	131	219	24
8	1941	647	129	32	388	97	162	18
9	1422	474	95	24	284	71	119	13
10	2094	698	140	35	419	105	175	19
11	1583	527	106	26	317	79	132	15
12	3648	1216	243	61	730	182	304	34
Mean ± SD	2144 ± 949	715 ± 316	143 ± 63	36 ± 16	429 ± 190	107 ± 47	177 ± 81	20 ± 9

Individual breakdown of energy and macronutrient intake for each test meal. Data were based on calorie intake from the Week 1 food diary analysis using Nutritics (Nutritics Academic Edition v4.267; Nutritics, Dublin, Ireland). Total intake was divided by 3 to calculate the lunch total. Lunch total was then reported as energy content of each macronutrient (kcals) with the meal composition consisting of 20% protein, 60% CHO, and 20% fat, with gram intake calculated using Atwater factors of 4, 4, and 9 kcal/g, respectively. CHO, carbohydrate.

**Table 3:** Breakdown of ingredients for each meal provided to participants.

Meal 1: White Wine and Garlic Sauce with Bread	Meal 2: Couscous Salad	Meal 3: Spaghetti with Tomato Sauce
Garlic White wine Onion Parsley Butter Bread Mussels	Couscous Garlic Cumin Tomatoes Cucumber Onion Olive oil Lemon Parsley Mussels	Spaghetti Garlic Onion White wine Olive oil Parsley Chopped Tomatoes Mussels
